# MicroRNA-566 activates EGFR signaling and its inhibition sensitizes glioblastoma cells to nimotuzumab

**DOI:** 10.1186/1476-4598-13-63

**Published:** 2014-03-20

**Authors:** Kai-Liang Zhang, Xuan Zhou, Lei Han, Lu-Yue Chen, Ling-Chao Chen, Zhen-Dong Shi, Ming Yang, Yu Ren, Jing-Xuan Yang, Thomas S Frank, Chuan-Bao Zhang, Jun-Xia Zhang, Pei-Yu Pu, Jian-Ning Zhang, Tao Jiang, Eric J Wagner, Min Li, Chun-Sheng Kang

**Affiliations:** 1 Department of Neurosurgery, Tianjin Medical University General Hospital; Laboratory of Neuro-Oncology, Tianjin Neurological Institute; Key Laboratory of Post-trauma Neuro-repair and Regeneration in Central Nervous System, Ministry of Education; Tianjin Key Laboratory of Injuries, Variations and Regeneration of Nervous System, Tianjin 300052, China; 2 The Vivian L. Smith Department of Neurosurgery, the University of Texas Medical School at Houston, Houston, TX 77030, USA; 3 The Department of Otorhinolaryngology and Maxillofacial Oncology, Tianjin Medical University Cancer Institute and Hospital; Key Laboratory of Cancer Prevention and Therapy, Tianjin Cancer Institute; National Clinical Research Center of Cancer, Tianjin 300060, China; 4 Department of Neurosurgery, The Second Affiliated Hospital of Harbin Medical University, Harbin 150086, China; 5 The State Key Laboratory of Experimental Hematology, Institute of Hematology and Blood Diseases Hospital, Chinese Academy of Medical Sciences and Peking Union Medical College, Tianjin 300020, China; 6 Tianjin Research Center of Basic Medical Science, Tianjin Medical University, Tianjin 300070, China; 7 Beijing Neurosurgical Institute, Department of Neurosurgery, Beijing Tiantan Hospital, Capital Medical University, 6 Tiantanxi Li, Beijing 100050, China; 8 Chinese Glioma Cooperative Group (CGCG), 6 Tiantanxi Li, Beijing 100050, China; 9 Department of Biochemistry and Molecular Biology, The University of Texas Medical School at Houston, Houston, TX 77030, USA

**Keywords:** EGFR, Glioblastoma, miR-566, Nimotuzumab, Combination therapy

## Abstract

**Background:**

Epidermal growth factor receptor (EGFR) is amplified in 40% of human glioblastomas. However, most glioblastoma patients respond poorly to anti-EGFR therapy. MicroRNAs can function as either oncogenes or tumor suppressor genes, and have been shown to play an important role in cancer cell proliferation, invasion and apoptosis. Whether microRNAs can impact the therapeutic effects of EGFR inhibitors in glioblastoma is unknown.

**Methods:**

miR-566 expression levels were detected in glioma cell lines, using real-time quantitative RT-PCR (qRT-PCR). Luciferase reporter assays and Western blots were used to validate VHL as a direct target gene of miR-566. Cell proliferation, invasion, cell cycle distribution and apoptosis were also examined to confirm whether miR-566 inhibition could sensitize anti-EGFR therapy.

**Results:**

In this study, we demonstrated that miR-566 is up-regulated in human glioma cell lines and inhibition of miR-566 decreased the activity of the EGFR pathway. Lentiviral mediated inhibition of miR-566 in glioblastoma cell lines significantly inhibited cell proliferation and invasion and led to cell cycle arrest in the G_0_/G_1_ phase. In addition, we identified von Hippel-Lindau (VHL) as a novel functional target of miR-566. VHL regulates the formation of the β-catenin/hypoxia-inducible factors-1α complex under miR-566 regulation.

**Conclusions:**

miR-566 activated EGFR signaling and its inhibition sensitized glioblastoma cells to anti-EGFR therapy.

## Background

Glioblastoma is the most common and fatal primary brain tumor in adults [1]. The survival time varies depending on the patient’s genetic background [2,3]. PTEN mutation and EGFR amplification are key prognostic factors in patients with anaplastic astrocytoma and in older patients with glioblastoma multiforme [4]. Molecular therapies targeting EGFR have been developed in recent years, such as gefitinib, but many patients do not respond well to EGFR inhibitors, including those with non-small-cell lung cancer or glioblastoma [5]. This is exemplified by the EGFR pathway’s contribution to radiation or chemo resistance in glioma [6].

MicroRNAs target the 3’ UTRs of oncogenes and tumor suppressor genes therefore contributing to the tumorigenesis of various human cancers [7]. We previously identified a group of microRNAs (miR-21, miR-23b, miR-27b and miR-524-5p) that regulate proliferation, invasion and apoptosis in glioma [8-11]. Additionally, we demonstrated that the expression profile of miR-566 as well as that of four other miRNAs (miR-181d, miR-518b, miR-524-5p and miR-1227) correlated with the prognosis of glioblastoma patients [12]. The function of miR-181d, miR-518, and miR-1227 have been reported in glioma or in other cancer types [13-15], however, there are no reports about miR-566 function till now.

Numerous studies have demonstrated that miRNAs contribute to chemotherapy resistance [16-18], most likely by regulating pro-survival pathways involved in drug resistance. Accumulating evidence suggests that microRNAs can regulate EGFR signaling, correlate with EGFR expression and influence gefitinib’s efficacy. For example, a study of lung cancer suggested that miRNA-128b directly regulated EGFR and loss of heterozygosity (LOH) was frequent in tumor samples, correlating significantly with the clinical response and survival following gefitinib treatment [19]. Furthermore, miR-21 repressed p53-mediated apoptosis in response to chemotherapeutic agents, such as doxorubicin and other DNA damage-inducing agents, thereby contributing to drug resistance in glioblastoma cells [20]. In this study, we focused on the function of miR-566 in EGFR signaling. We hypothesized that miR-566 could regulate the EGFR pathway and influence the sensitivity of glioma cells to anti-EGFR therapy.

## Results

### miR-566 is over-expressed in glioma cell lines and activates EGFR/Akt signaling

**Figure 1:**
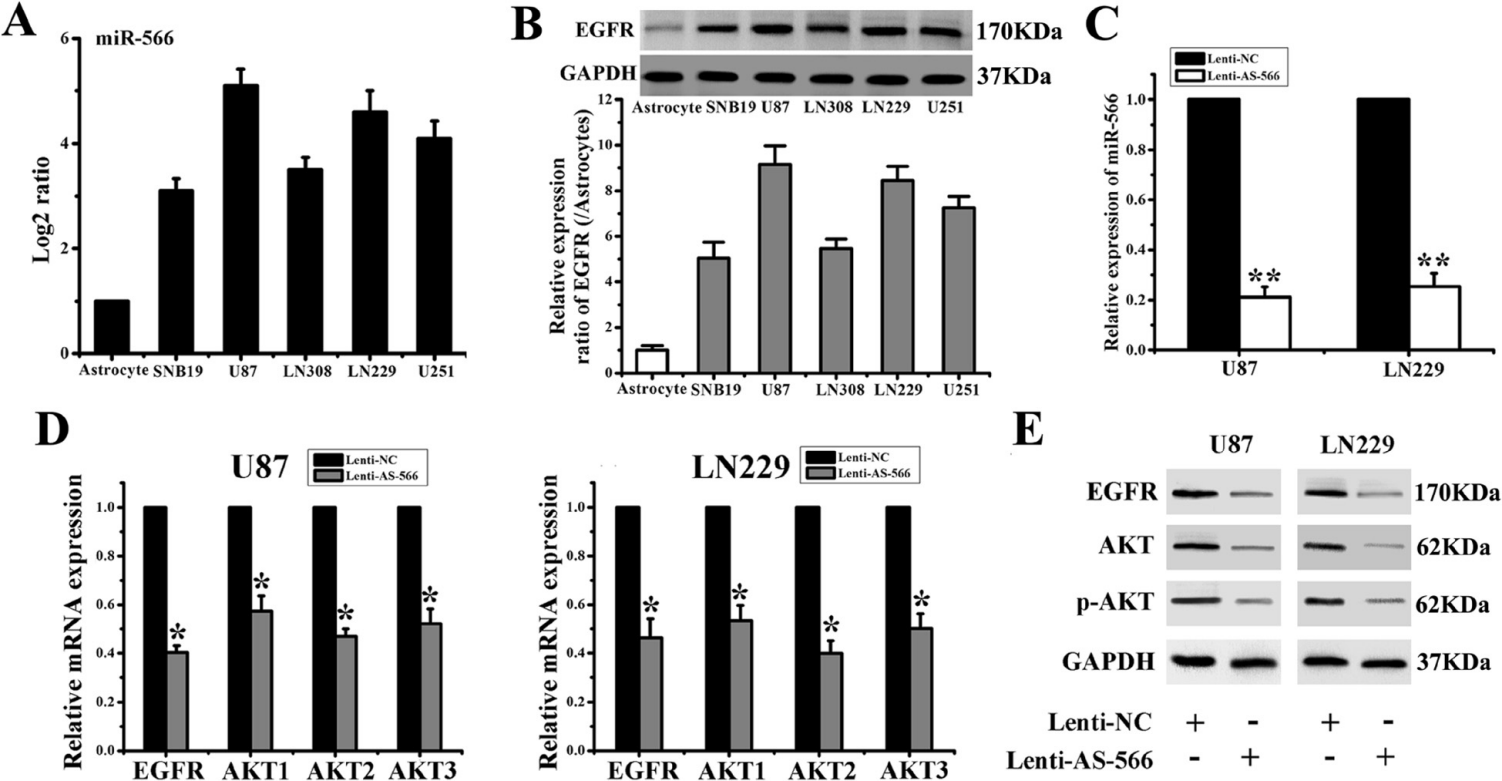
**miR-566 inhibitor blocked EGFR/Akt signaling. (A)** qRT-PCR analysis of miR-566 in total RNA from indicated glioma cell lines and normal astrocytes. The data shown are average fold changes (the mean ± s.e. of 3 individual experiments) of individual miR-566 expression in each cell line relative to astrocytes. **(B)** Western blot (top) and real-time RT-PCR (bottom) were used to detect the expression of EGFR in a panel of glioma cell lines and astrocytes. GAPDH was used to normalize the initial input of total RNA. The expression levels of the transcripts of EGFR were based on the amount of target message relative to GAPDH. Bar graphs show the ratio of the expression level in each cell line to that in astrocytes. **(C)** qPCR was used to measure the expression of miR-566 in glioma cells that were infected with or without lenti-AS-566. The data are shown as the mean ± SD. **, *P* < 0.01. **(D, E)** U87 and LN229 glioma cells were either infected with lenti-NC or lenti-AS-566, and 48 h later, real-time RT-PCR **(D)** and Western blot **(E)** were used to detect the expression of EGFR and Akt.

We previously demonstrated that the expression of five microRNAs (miR-181d, miR-518b, miR-524-5p, miR-566 and miR-1227) were correlated with the survival of glioblastoma patients [12]. Previous studies have clarified the functions of the miR-181 family, miR-518b, miR-524-5p and miR-1227. However, the function of miR-566 has not been reported. To address the function of miR-566, we first examined the expression of miR-566 in a panel of five glioma cell lines and normal astrocytes by qPCR. The results showed that miR-566 was overexpressed in all the glioma cell lines, especially in U87 and LN229 cells. The expression level of miR-566 was 5.1 fold in U87 and 4.7 fold in LN229 cells than miR-566 in control astrocytes (Figure 1A). We selected these two cell lines for further functional analysis and examined the expression of EGFR at both the mRNA and protein levels. Interestingly, the same expression profile was detected in five glioma cell lines, including normal astrocytes (Figure 1B). Therefore, we hypothesized that the EGFR pathway maybe regulated by miR-566. To confirm this, a lentivirus containing the miR-566 inhibitor segment was constructed to down-regulate the expression of miR-566. qPCR analysis indicated that the lentivirus could successfully reduce miR-566 expression by 79% and 75% in U87 and LN229 cells, respectively (Figure 1C). Finally, EGFR and Akt expression were detected both at the mRNA and protein levels, which revealed that the miR-566 inhibitor deactivated EGFR/Akt signaling (Figure 1D and E).

### Inhibition of miR-566 inhibits proliferation and invasiveness of glioma cells

**Figure 2:**
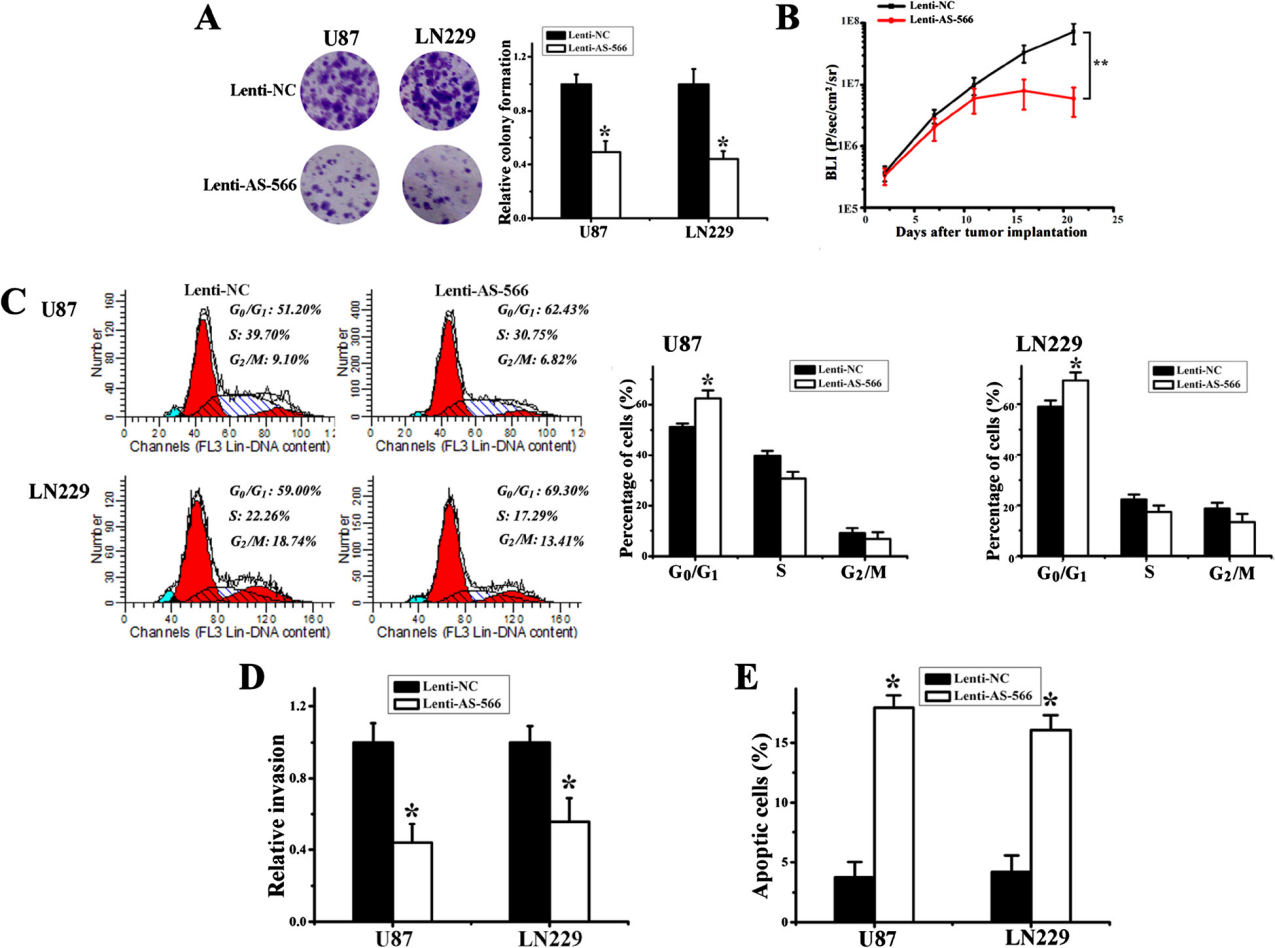
**miR-566 inhibitor suppressed the proliferation and invasive potential of glioma cells. (A)** U87 and LN229 glioma cells were treated with lenti-NC or lenti-AS-566. After 24 h, viable cells were harvested and reseeded at 2000 cells/well onto 6-well plates as a single-cell suspension. Cells were allowed to grow for 14 days before fixing with methanol and staining with crystal violet. The data shown are representative of 3 individual experiments. **(B)** Mice were implanted with U87 cells pretreated with a luciferase reporter containing lenti-virus and lenti-NC or lenti-AS-566. Tumor mass was determined by bioluminescence at day 3, 7, 11, 16 and 21. The data are shown as the mean ± SD. **, *P* < 0.01. **(C-E)** U87 and LN229 glioma cells were infected with lenti-NC or lenti-AS-566. After 48 h, the cell cycle distribution **(C)** and apoptosis **(E)** were detected. Cell invasion was analyzed 24 h after seeding in transwells **(D)**.

After demonstrating that miR-566 deactivates the EGFR/Akt pathway, we examined miR-566’s function in glioma cell proliferation, invasion, apoptosis and cell cycle distribution. We investigated the effects of miR-566 inhibition on the growth and viability of U87 and LN229 glioma cells. Lenti-AS-566 significantly suppressed the colony forming abilities of these cells (to 47% and 39%, respectively; Figure 2A) compared to lenti-NC treated cells. To examine the potential role of miR-566 in tumorigenesis, we infected U87 cells with lenti-AS-566 or lenti-NC and used these cells in an intracranial model, and tumor size was assessed weekly by a luciferase bioluminescence imaging system. Notably, the inhibition of miR-566 expression in U87 cells resulted in delayed tumor formation and a dramatic reduction in tumor size compared to the lenti-NC group (Figure 2B). This result demonstrated that miR-566 inhibits the tumorigenicity of glioma cells both *in vitro* and *in vivo*. We then investigated how miR-566 affects the invasive and apoptotic behaviors of glioma cells. U87 and LN229 cells were infected with lenti-AS-566, and 48 h after infection, a cell invasion assay was performed. Significantly increased G_1_ cell cycle arrest was observed in lenti-AS-566-infected cells (Figure 2C). We also found a significant decrease in cell invasion (Figure 2D) compared to lenti-NC-treated cells. Apoptosis and cell cycle analysis based on flow cytometry were performed with infected glioma cells (U87 and LN229). A significant increase in the number of apoptotic cells in lenti-AS-566-infected cells (U87 and LN229) was also detected (Figure 2E). miR-566 inhibitor had no effect on the apoptosis of normal astrocytes (Additional file 1: Figure S1).

Having confirmed that a miR-566 inhibitor could deactivate the EGFR pathway and inhibit the proliferative and invasive behavior of glioma cells, we then demonstrated whether the functions of miR-566 were mainly through the EGFR pathway. U87 glioma cells were first infected with lenti-AS-566 and then EGF was introduced to activate EGFR signaling. Results showed that EGF partially reversed the effects of lenti-AS-566 (Additional file 1: Figure S2).

### VHL is a direct target of miR-566

**Figure 3:**
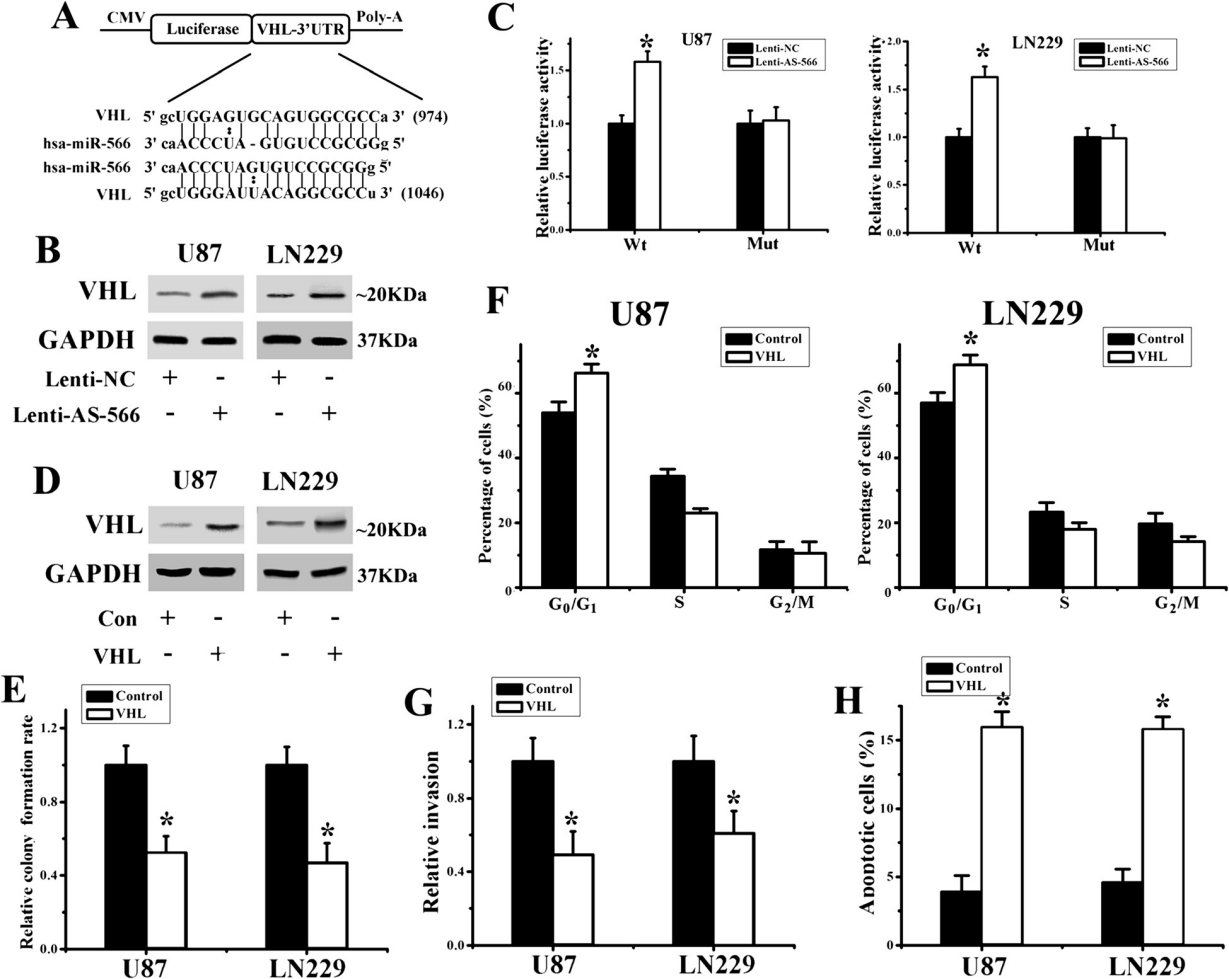
**VHL is a direct functional target of miR-566. (A)** A 3’ UTR fragment containing the predicted miR-566 targeting sites of VHL were fused downstream of the firefly luciferase gene. **(B)** Western blot analysis of VHL protein expression in U87 and LN229 cells treated with lenti-NC or lenti-AS-566. **(C)** U87 and LN229 glioma cells were co-transfected with a human VHL 3’ UTR firefly luciferase reporter plasmid and lenti-NC or lenti-AS-566 as indicated. After 48 h, firefly luciferase activity was measured. **(D)** Western blot was used to detect the expression of the VHL protein 48 h after transfection of the VHL expression plasmid or control plasmid. **(E-H)** Overexpression of VHL rescued the effect of miR-566 on glioma cell proliferation **(E)**, cell cycle distribution **(F)**, invasion **(G)** and apoptosis **(H)**. *P < 0.05.

We have clarified that a miR-566 inhibitor blocks proliferation and invasion and accelerates apoptosis in glioma cells partially through the EGFR pathway. We then further confirmed the target of miR-566. VHL was shown to be involved in the tumorigenesis of glioma [9,21], and we found that the 3’ UTR of VHL contained 2 potential complimentary binding sites for miR-566. To validate this, we performed luciferase reporter assays using 3’ UTR sequence fragments of the VHL transcript containing the two predicted binding sites for miR-566 (Figure 3A). Western blot analysis demonstrated that VHL protein expression was significantly increased in lenti-AS-566-infected glioma cells (Figure 3B). Transient cotransfection of human U87 and LN229 cells with lenti-AS-566 and a wild-type VHL 3’ UTR plasmid led to a significant increase in luciferase reporter activity compared to infection with lenti-NC (Figure 3C). ‘Rescue’-experiments were performed by introducing a VHL expression plasmid lacking the miR-566 3’ UTR region into U87 and LN229 glioma cells to confirm that the growth and invasion inhibition, cell cycle arrest and apoptosis promotion by lenti-AS-566 was mediated, at least in part, through the induction of VHL expression. The successful transfection of the VHL plasmid was monitored at the VHL protein level (Figure 3D). We then performed colony formation assays, invasion assays and flow cytometry in VHL plasmid-transfected glioma cells. As shown in Figure 3E and G, cell proliferation and invasion activity were significantly inhibited in VHL-transfected glioma cells (48 h after transfection). As shown in Figure 3 F and H, G_1_ cell cycle arrest was induced and apoptosis was increased in VHL-transfected glioma cells.

### miR-566 regulates the EGFR pathway through the VHL/β-catenin and VHL/HIF-1α axis

**Figure 4:**
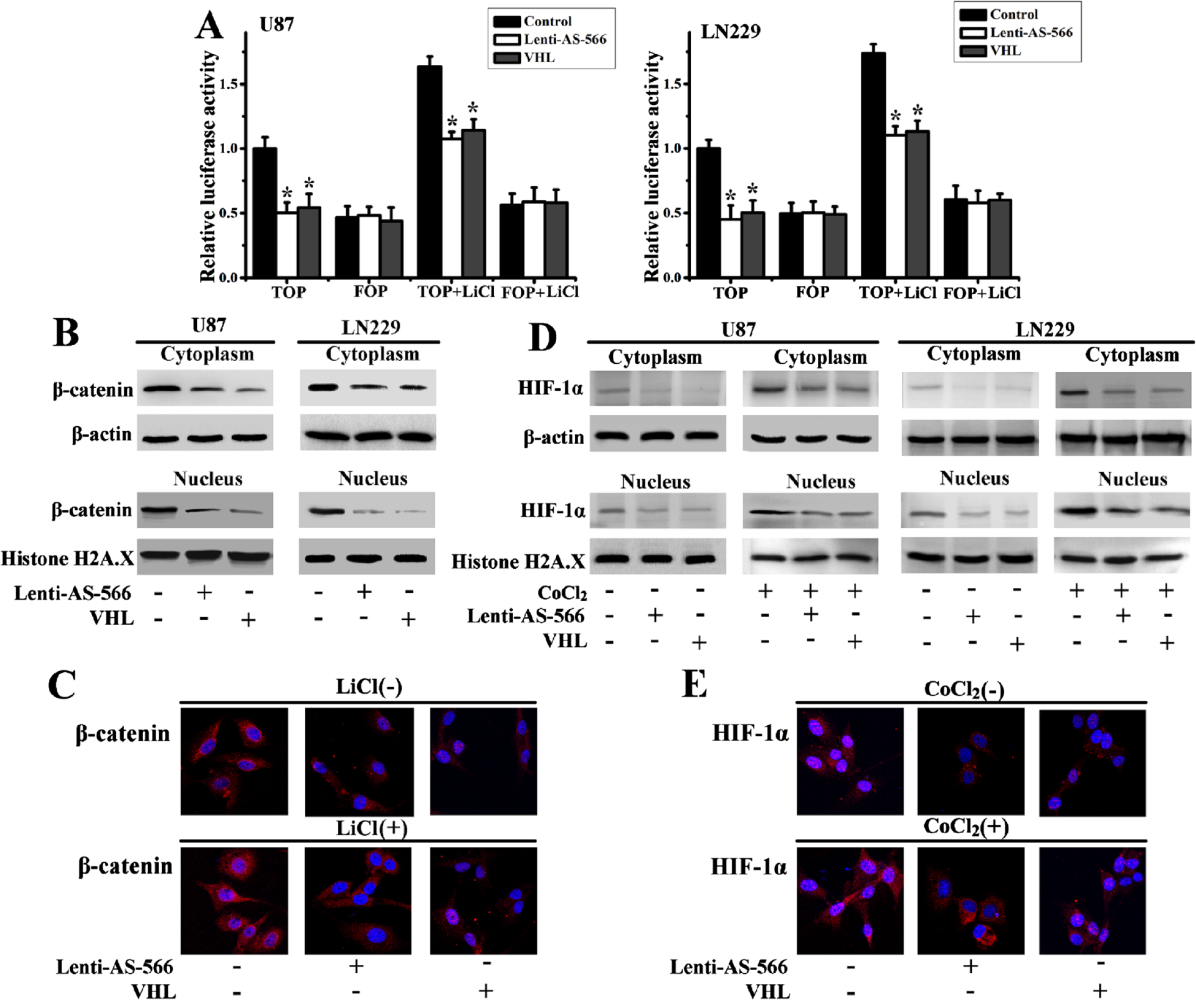
**miR-566 regulated the β-catenin and HIF-1α pathway through VHL. (A)** U87 and LN229 cells were treated with lenti-AS-566 or VHL plasmid, and TOPFLASH activity was measured. The data are shown as the mean ± SD of 3 independent experiments. *, *P* < 0.05, compared with vector control. **(B, D)** Cytoplasmic and nuclear proteins were prepared from lenti-AS-566 or VHL plasmid treated U87 and LN229 cells with or without the addition of CoCl_2_. Cytoplasmic and nuclear β-catenin and HIF-1α expression levels were detected. β-actin and Histone H2A.X were used as loading controls for cytoplasmic and nuclear proteins, respectively. **(C, E)** U87 cells were infected with lenti-AS-566 or VHL plasmid in the presence of LiCl, CoCl_2_ or vehicle for 24 h. Confocal images show the translocation of β-catenin and HIF-1α in U87 cells. DAPI was used to stain the nuclei. Bar, 10 μm.

TOP/FOP FLASH reporter and luciferase constructs were introduced to detect the activity of canonical Wnt signaling. TOPFLASH activity was measured in U87 and LN229 cells infected with lenti-AS-566 or transfected with VHL plasmid in the presence of LiCl or not. Our Results showed that miR-566 inhibition or VHL overexpression could reduce the activity of the β-catenin/TCF4 pathway (Figure 4A). Western blot was used to examine the expression of β-catenin both in the cytoplasm and nucleus. We found that lenti-AS-566 or VHL plasmid decreased the expression of β-catenin, especially in the nucleus (Figure 4B). Confocal images showed the same results (Figure 4C). Together, these findings suggested that miR-566 regulated the activity of β-catenin/TCF signaling through targeting VHL. The same results were found in the HIF-1α pathway (Figure 4D and E). All together, we demonstrated that miR-566 could regulate both β-catenin and HIF-1α signaling by targeting VHL.

### miR-566 regulates the formation of the β-catenin/HIF-1α complex and sensitizes glioma cells to nimotuzumab therapy

**Figures 5:**
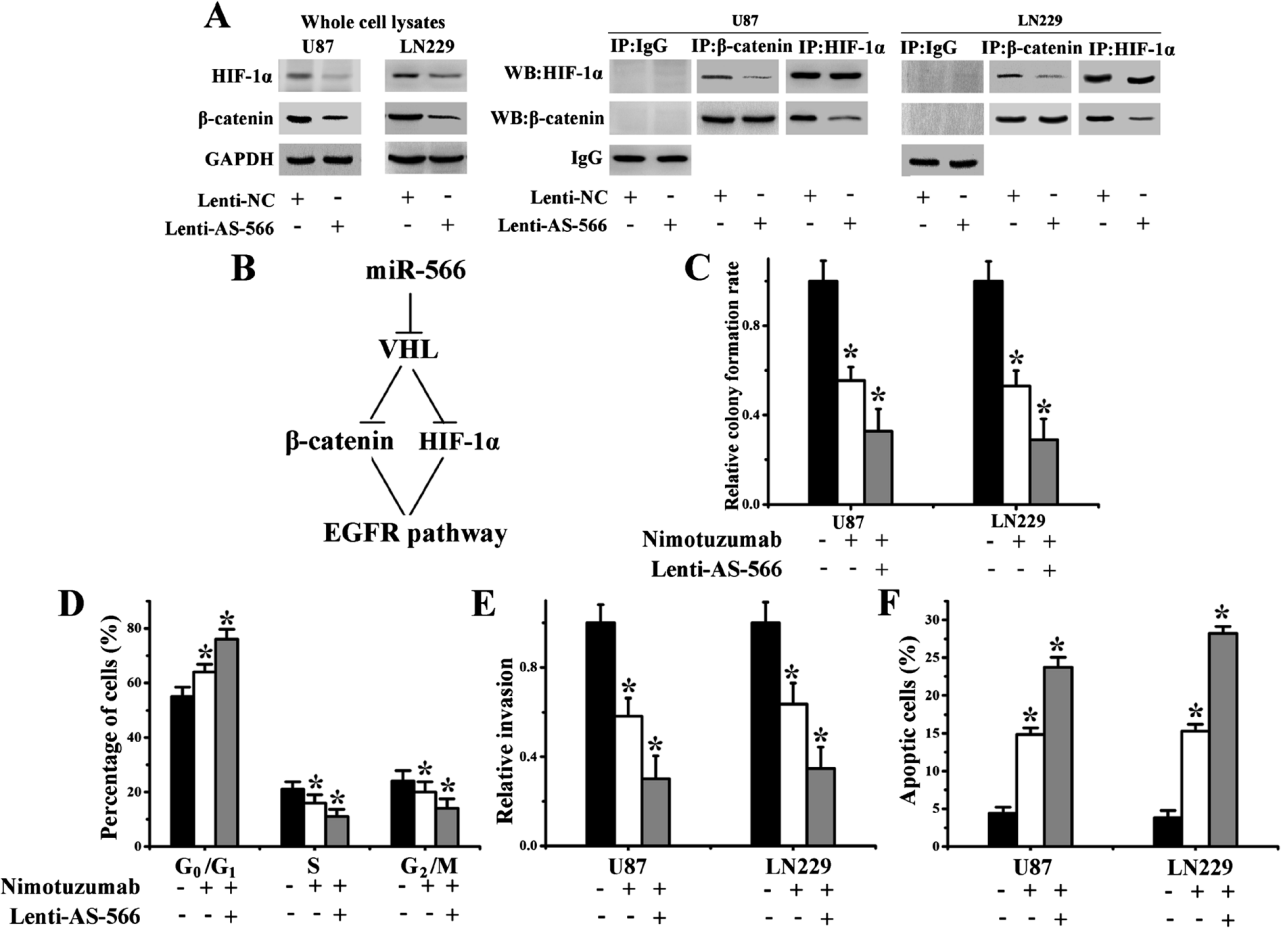
**miR-566 regulated the formation of the β-catenin/HIF-1α complex and sensitized glioma cells to nimotuzumab therapy. (A)** Freshly isolated cell lysates (U87 and LN229 either infected with lenti-AS-566 or left untreated) were used to immunoprecipitate β-catenin or HIF-1α with specific rabbit antibodies. Rabbit whole immunoglobulin (IgG) was used as a control antibody for immunoprecipitation assays. The immunoprecipitated complexes were subjected to Western blot analysis with specific antibodies against β-catenin and HIF-1α as indicated. **(B)** A schematic diagram showed the miR-566 modulated EGFR pathway through VHL/β-catenin/HIF-1α. **(C-F)** U87 and LN229 cells were treated with nimotuzumab (100 μg/ml) as indicated, and after 24 h, cells were infected with lenti-AS-566 or were untreated. Cell proliferation **(C)**, cell cycle distribution **(D)**, *in vitro* invasion **(E)** and apoptosis **(F)** were evaluated 4 d after lentiviral infection. The data in all panels represent the mean ± SD. *, *P* < 0.05.

VHL is responsible for the degradation of β-catenin and HIF-1α [22], transcription factors that regulate the expression of EGFR [23,24]. Therefore, we examined whether HIF-1α forms a complex with β-catenin in glioma cells by performing co-IP experiments. The results showed that HIF-1α forms a complex with β-catenin (Figure 5A). Furthermore, lenti-AS-566 partially inhibited the formation of this complex. Western blot analysis was used to determine the endogenous expression levels of HIF-1α or β-catenin in the glioma cell lysates used in these experiments (Figure 5A). These results suggested that HIF-1α and β-catenin form a complex and cooperatively promote EGFR synthesis (Figure 5B).

### Synergistic activity of miR-566 inhibition and nimotuzumab in glioma cells and xenograft model

**Figure 6:**
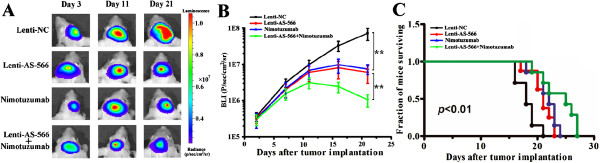
**Lenti-AS-566 sensitized glioma cells to the effects of nimotuzumab in a mice xenograft model. (A)** Bioluminescence images from nimotuzumab and/or lenti-AS-566-treated animals at 3, 11 and 21 d after tumor implantation. **(B)** Tumor growth curves were evaluated. The data are shown as the mean ± SD. **, *P* < 0.01. **(C)** Improved survival was observed in mice that were treated with the combined therapy.

We have shown that miR-566 is an important oncomiR in EGFR pathway regulation. We then determined if miR-566 inhibition has synergistic effects with nimotuzumab administration. U87 and LN229 cells were treated with nimotuzumab (100 μg/ml), and after 24 h, infected with lenti-AS-566 or mock control. Cell proliferation (Figure 5C), cell cycle distribution (Figure 5D), *in vitro* invasion (Figure 5E) and apoptosis (Figure 5 F) were evaluated four days after-lentiviral infection. Lenti-AS-566 enhanced the effects of nimotuzumab with suppression of cellular proliferation and invasion (Figure 5C and E). Flow cytometric analysis revealed that more cells were arrested in the G_1_ phase in the combination group (Figure 5D). In addition, more apoptotic cells were detected after treatment with nimotuzumab combined with lenti-AS-566 (Figure 5 F). To evaluate the effects of the combined therapy of nimotuzumab and miR-566 inhibition on tumor growth *in vivo*, we established tumors as intracranial xenografts in nude mice. U87 cells were pretreated with lentivirus containing a luciferase reporter. Compared with nimotuzumab alone, the combination of nimotuzumab with lenti-AS-566 significantly decreased the tumor burden (Figure 6A and B). To analyze the survival times of the treatment groups, we generated Kaplan-Meier survival curves (Figure 6C), which demonstrated that combined therapy significantly prolonged survival.

## Discussion

To our knowledge, there are no reports on the function of miR-566 in human cancers including glioma. In the present study, we confirmed that miR-566 was upregulated in human glioma cells, and repressing miR-566 could inactivate the EGFR pathway largely by targeting VHL. Further studies demonstrated that miR-566 regulated the VHL/β-catenin and VHL/HIF-1α axis in the transcription of EGFR. In addition, miR-566 is responsible for the formation of a β-catenin/HIF-1α complex. Finally, we confirmed that miR-566 inhibition could be synergistic with nimotuzumab therapy.

The EGFR pathway is activated in glioma and other human cancers, including lung, breast and colorectal [25-28]. The FDA has approved two types of anti-EGFR agents: low molecular weight tyrosine kinase inhibitors (TKIs) and mAbs that inhibit the EGFR extracellular domain. Clinically used anti-EGFR drugs include gefitinib, erlotinib, lapatinib, cetuximab and panitumumab [29,30]. TKIs are effective therapies in human non-small cell lung cancer [31,32]. However, TKIs such as gefitinib and erlotinib have had limited clinical success in treating glioblastoma [33]. Moreover, all patients treated with cetuximab or panitumumab for colorectal cancer suffered from acute and subacute cutaneous side effects [34]. Nimotuzumab is an anti-EGFR mAb developed at the Center of Molecular Immunology in Havana, Cuba. The clinical trials of nimotuzumab demonstrated that severe cutaneous adverse events were extremely rare. Furthermore, grade 3 and 4 acneiform eruptions commonly associated with other anti-EGFR mAbs were absent [35]. Glioblastoma patients could benefit from nimotuzumab therapy, but the molecular expression profiles of GBM patients differ from one another. Personalized and combination therapy are needed.

MiRNAs are small, non-coding RNAs that can function as oncogenes or tumor suppressors by inhibiting the expression of numerous target genes. EGFR signaling can also be regulated by numerous miRNAs. For example, miR-7 is down-regulated in human glioblastoma and directly inhibits EGFR expression by targeting its 3’ UTR. In addition, miR-7 suppresses Akt pathway activation independent of its EGFR inhibition [36]. We previously demonstrated that miR-21 is upregulated in glioma cells and that blocking its expression inactivates EGFR/Akt signaling in a PTEN-independent manner [8]. In the present study, for the first time, we confirmed that miR-566 is upregulated in human glioma cells. *In vitro* and *in vivo* studies demonstrated that miR-566 inhibition deactivated EGFR/Akt signaling and slowed the proliferation of glioma cells.

Studies have demonstrated that miRNAs influence the response to chemotherapies for ovarian cancer, pancreatic cancer, bladder cancer and glioblastoma [37-40]. In a study conducted by Liana Adam, miR-200 expression regulated the epithelial-to-mesenchymal transition in bladder cancer cells and reversed EGFR therapy resistance [41]. In a study by Masahiro Seike, miR-21 was up-regulated in the lung adenocarcinoma cell line H3255, which contains an EGFR mutation and is hypersensitive to EGFR TKI AG1478. The inhibition of miR-21 enhanced AG1478-induced apoptotic activity in these lung cancer cells, which showed intermediate sensitivity to AG1478. Another study demonstrated that epidermal growth factor (EGF) and MET receptors modulated the expression of miR-30b, miR-30c, miR-221 and miR-222. These microRNAs are also responsible for gefitinib-induced apoptosis and the epithelial-mesenchymal transition of NSCLC cells *in vitro* and *in vivo* by inhibiting the expression of the genes encoding BCL2-like 11 (BIM), apoptotic peptidase activating factor 1 (APAF-1), protein kinase C ϵ (PKC-ϵ) and sarcoma viral oncogene homolog (SRC) [42]. Our previous data demonstrated that miR-21 is involved in the regulation of anti-EGFR therapy [43].

Because miR-566 can regulate EGFR signaling, we wondered whether it could sensitize glioma to the effects of nimotuzumab *in vitro* and *in vivo* and its underlying mechanism. We identified VHL as a potential functional target of miR-566. A 3’ UTR luciferase assay was performed to determine whether miR-566 binds to the 3’ UTR of the VHL gene. The relative luciferase level for the VHL gene was significantly higher in lenti-AS-566-infected glioma cells than in lenti-NC-infected controls, and Western blot analysis confirmed these findings. The results demonstrated that the expression of the VHL protein is significantly upregulated in lenti-AS-566 infected cells. These results suggest that VHL is a direct target of miR-566. Furthermore, we confirmed that miR-566 regulated the formation of a β-catenin/HIF-1α complex. Both β-catenin and HIF-1α are important transcription factors for EGFR. Finally, studies demonstrated that the proliferation and invasion of glioma cells are attenuated when co-treated with lenti-AS-566 and nimotuzumab. The same results were confirmed in nude mice treated with lenti-AS-566 and nimotuzumab.

## Conclusions

In conclusion, this is the first report to demonstrate that miR-566 expression is significantly increased in glioma cells. miR-566 modulated the EGFR pathway through direct targeting of VHL. We have identified the survival-related miRNA miR-566 as a regulator that influences the response to anti-EGFR therapy. Our study could have important implications for glioblastoma patients in the development of novel therapeutics.

## Materials and methods

### Cell culture and chemical reagents

The human glioma cell lines U87, LN229, SNB19, LN308 and U251 were obtained from the American Type Culture Collection (ATCC, Manassas, VA, USA). Human astrocytes (Invitrogen, Carlsbad, CA) were derived from human brain tissues. The human glioma cell lines were cultured in Dulbecco’s modified Eagle medium (DMEM) supplemented with 10% heat-inactivated fetal bovine serum (FBS, Hyclone, Waltham, MA). Astrocytes were cultured in GIBCO Astrocyte Medium supplemented with N-2, FBS and EGF. Cells were cultured in a humidified 10% CO_2_ atmosphere at 37˚C. LiCl (Acros Organics, New Jersey, USA) and CoCl_2_ (Sinopharm Chemical, Shanghai, China) were diluted in phosphate-buffered saline (PBS).

### Lentiviral infection, gene transfection and qRT-PCR

Lentiviruses containing a miR-566 inhibitor segment (lenti-AS-566) or negative control (lenti-NC) segment were obtained from Genepharma (Shanghai, China). The human glioma cell lines U87 and LN229 were infected with the viral suspension. pcDNA3 and pcDNA3-VHL plasmids were transfected using Lipofectamine 2000 (Invitrogen, Carlsbad, CA) following the manufacturer’s instructions. Cells were harvested 48 h after infection or transfection, and RNA and protein extractions were performed. TRIzol (Invitrogen, Carlsbad, California) was used to isolate total RNA. To detect miR-566, stem-loop reverse transcription-polymerase chain reaction (RT-PCR) was performed with a one-step RNA PCR kit (Takara, Otsu, Shiga, Japan) according to the manufacturer’s instructions. Real-time PCR was performed by SYBR green detection with a forward primer for the mature miRNA sequence and a universal adaptor reverse primer. For the analysis of *EGFR*, *AKT1*, *AKT2* and *AKT3* messenger RNA (mRNA) expression, complementary DNA (cDNA) synthesis was performed using random primers under standard conditions. mRNA expression was quantified using the ΔΔCt method. *GAPDH* served as the internal control. All miRNA expression data were normalized to a U6 small nuclear RNA from the same sample. All reactions were performed in triplicate.

### Plasmid construction and 3’ UTR analysis

The VHL expression plasmid pcDNA3-VHL was kindly provided by Professor Jinquan Cheng (H. Lee Moffitt Cancer Center and Research Institute, Florida). Glioma cells were transfected with 100 ng TOP-FLASH or FOP-FLASH plasmid (Millipore, Billerica, Massachusetts). The cells were then treated with lenti-AS-566 or VHL plasmid with or without LiCl. At 24 h after transfection, cell lysates were prepared with Dual Luciferase Lysis Buffer (Promega, Agora, Fitchburg Center, Fitchburg, Wisconsin), and luciferase activity was measured with a microplate reader (Mithras LB940; Berthold Technologies GmbH, Bad Wildbad, Germany). The transfection efficiency was normalized using *Renilla* luciferase activity. Experiments were performed at least 3 times; representative data from a single experiment are shown.

The putative miR-566 binding site of the VHL 3’ UTR was inserted into the pGL3-control vector (Promega, Agora, Fitchburg Center, Fitchburg, Wisconsin) at the *Xba I* site. For the VHL mutant reporter, the seed region of the VHL 3’ UTR was deleted to remove all nucleotides with complementarity to miR-566. For 3’ UTR luciferase assays, glioma cells were co-treated with lenti-NC or lenti-AS-566. Luciferase assays were performed using the Dual-Luciferase Reporter Assay System (Promega, Agora, Fitchburg Center, Fitchburg, Wisconsin) 48 h after transfection.

### Protein extraction, immunoblotting and immunoprecipitation

Glioma cells were washed in PBS and lysed with ice-cold RIPA buffer (Pierce, Brebieres, France) containing the protease inhibitor PMSF (Sigma, St. Louis, MO). Protein quantification was performed with a NanoDrop ND-1000 Spectrophotometer (NanoDrop Technologies, Wilmington, USA). A DUALXtract Cytoplasmic and Nuclear Protein Extraction Kit (Dualsystems Biotech, Schlieren, Switzerland) was used to isolate cytoplasmic and nuclear proteins from cultured glioma cells.

The protein lysates were subjected to sodium dodecyl sulfate-polyacrylamide gel electrophoresis (SDS-PAGE) and transferred to a polyvinylidene difluoride membrane (Roche, Basel, Switzerland). Membranes were immunostained with specific antibodies according to standard protocols. Antibody-labeled protein bands on the membranes were detected with a G:BOX F3 (Syngene, Cambridge, UK).

For immunoprecipitation, cells were lysed with IP lysis buffer (Pierce, Rockford, USA). The cell lysates were then subjected to immunoprecipitation with 1–5 mg of antibodies and Protein A/G agarose beads (Pierce, Rockford, USA) overnight at 4°C with constant agitation. Control samples were incubated with agarose beads after immunoprecipitation with a control immunoglobulin. The immunoprecipitated complexes were then washed with wash buffer. The proteins were eluted, boiled and subjected to SDS-PAGE analysis.

### Immunofluorescence analysis

For immunofluorescence analysis, glioma cells were seeded on poly-L-Lysine treated coverslips (BD, USA). The cells were then infected with lenti-566 or lenti-NC with or without LiCl or CoCl_2_. After 48 h, the cells were fixed in cold methanol for 2 min. The cells were then washed 3 times in PBS and incubated in blocking buffer for 30 min at room temperature. Next, the cells were washed in PBS and incubated overnight at 4°C with β-catenin and HIF-1α primary antibodies (Cell Signaling Technology). The cells were again washed in PBS, followed by incubation with a fluorescent secondary antibody for 1 h at room temperature. Nuclei were stained with DAPI solution for 5 min. Confocal images of the cells were acquired on a confocal microscope (FV500) with a 40 × water immersion lens and a 1.20 numerical aperture using FluoView software (Olympus, Japan).

### Colony formation, invasion, cell cycle distribution and apoptosis analysis

For the colony formation assay, 2,000 glioma cells treated with lenti-AS-566, lenti-NC, VHL plasmid or nimotuzumab were plated in complete growth media in a fresh 6-well plate and allowed to grow until visible colonies formed. Cold methanol was used to fix the cell colonies, and colonies were stained with 0.1% crystal violet for 15 min, washed, air dried, photographed and counted.

Corning transwell insert chambers (Corning, New York) and BD Matrigel Invasion Chambers (BD Biosciences, Bedford, MA) were used for the cell invasion experiment. The prepared cells were added to the chamber and incubated for 24 h at 37˚C. Cells that invaded the lower chamber through the membrane were fixed with 20% methanol and stained with 0.1% crystal violet, imaged and counted.

The cell cycle was analyzed by flow cytometry. Pretreated U87 and LN229 cells were washed with PBS, trypsinized, fixed in 70% ethanol, washed and incubated in phosphate-buffered saline containing propidium iodide and RNase A (Sigma, St. Louis, MO) for 30 min at 37˚C. The cell cycle distributions were determined using a DNA stain (4’,6-diamidino-2-phenylindole). The data are the mean ± SD of 3 independent experiments.

Forty-eight hours after transfection, cells were harvested, washed, resuspended in staining buffer and examined using an Annexin V FITC Apoptosis Detection Kit (KeyGEN Biotech, Nanjing, China). The apoptotic distribution of the cells in each sample was then determined using fluorescence-activated cell sorting. Annexin V-positive cells were regarded as apoptotic cells.

### Intracranial model

Athymic mice (4 weeks of age) were intracranially implanted with 5 × 10^5^ U87 cells (pretreated with lentivirus containing the miR-566 inhibitor segment or negative control segment) under the direction of a stereotactic instrument. Four days after cell implantation, mice were injected intraperitoneally with nimotuzumab or control PBS every other day. Bioluminescence imaging was used to detect intracranial tumor growth. Mice were anesthetized, injected with D-luciferin (Promega, Agora, Fitchburg Center, Fitchburg, Wisconsin) at 50 mg/mL intraperitoneally and imaged with the IVIS Imaging System (Caliper Life Sciences) for 10–120 s. To quantify bioluminescence, identical circular regions of interest were drawn around the entire head of each animal, and the integrated flux of photons (photons per second) in each region of interest was determined by using the Living Images software package (Caliper Life Sciences). Data were normalized to the bioluminescence at the initiation of treatment for each animal. The error bars shown in the figures indicate SDs. All protocols involving animals were performed in accordance with an approved Institutional Animal Care and Use Committee protocol.

## Competing interest

The authors declare that they have no competing interests.

## Authors’ contributions

Conception and design: CSK, ML, KLZ, XZ; Development of methodology: KLZ, XZ, LH, LYC, LCC, ZDS; Acquisition of data (provision of animals, acquisition, provision of facilities, etc.): KLZ, XZ, LH, MY, YR, CBZ, JXZ, JNZ, PYP; Writing, reviewing, and/or revision of the manuscript: ML, CSK, EJW, KLZ, XZ, TSF, JXY; Study supervision: CSK, ML, PYP, JNZ, TJ. All authors read and approved the final manuscript.

## Supplementary Material

Additional file 1: Figure S1 miR-566 inhibitor had no effect on the apoptosis of normal astrocytes. **(A)**, Astrocytes were infected or not with lenti-AS-566. After 48 h, apoptosis was detected. **(B)**, Western blot was used to examine the expression of Bcl-2 in astrocytes. **Figure S2.** EGF reversed the effects of miR-566 inhibition. (A) U87 cells were infected or not with lenti-AS-566, 24 h later, EGF (10 ng/ml) was added into the medium. Proliferation **(A)**, cell cycle distribution **(B)**, *in vitro* invasion **(C)**, and caspase3/7 activity **(D)** were evaluated 10 h after EGF treatment. Data in all panels represent the mean ± SD. *, P < 0.05; **, P < 0.01. Click here for file
