# Supplementary material for: MicroRNA-566 activates EGFR signaling and its inhibition sensitizes glioblastoma cells to nimotuzumab
Source: Mol Cancer. 2014 Mar 20;13:63. doi: 10.1186/1476-4598-13-63 (PMC3999939; doi:10.1186/1476-4598-13-63)
Supplement: Additional file 1: Figure S1 — miR-566 inhibitor had no effect on the apoptosis of normal astrocytes. (A), Astrocytes were infected or not with lenti-AS-566. After 48 h, apoptosis was detected. (B), Western blot was used to examine the expression of Bcl-2 in astrocytes. Figure S2. EGF reversed the effects of miR-566 inhibition. (A) U87 cells were infected or not with lenti-AS-566, 24 h later, EGF (10 ng/ml) was added into the medium. Proliferation (A), cell cycle distribution (B), in vitro invasion (C), and caspase3/7 activity (D) were evaluated 10 h after EGF treatment. Data in all panels represent the mean ± SD. *, P < 0.05; **, P < 0.01. [file 1476-4598-13-63-S1.docx]

**
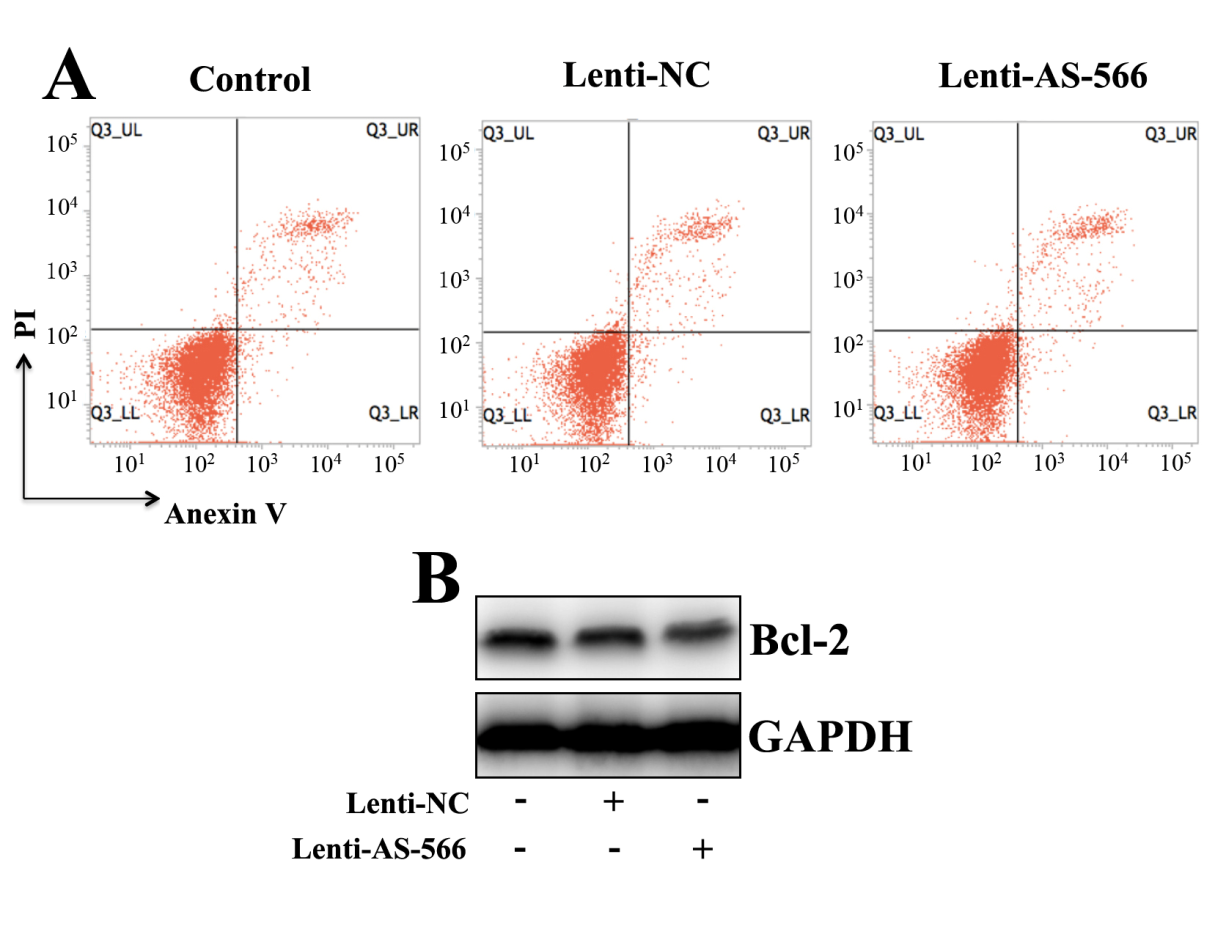
Additional file 1: Figure S1 – miR-566 inhibitor had no effect on the apoptosis of normal astrocytes. (A)**, Astrocytes were infected or not with lenti-AS-566. After 48 h, apoptosis was detected. **(B)**, Western blot was used to examine the expression of Bcl-2 in astrocytes.


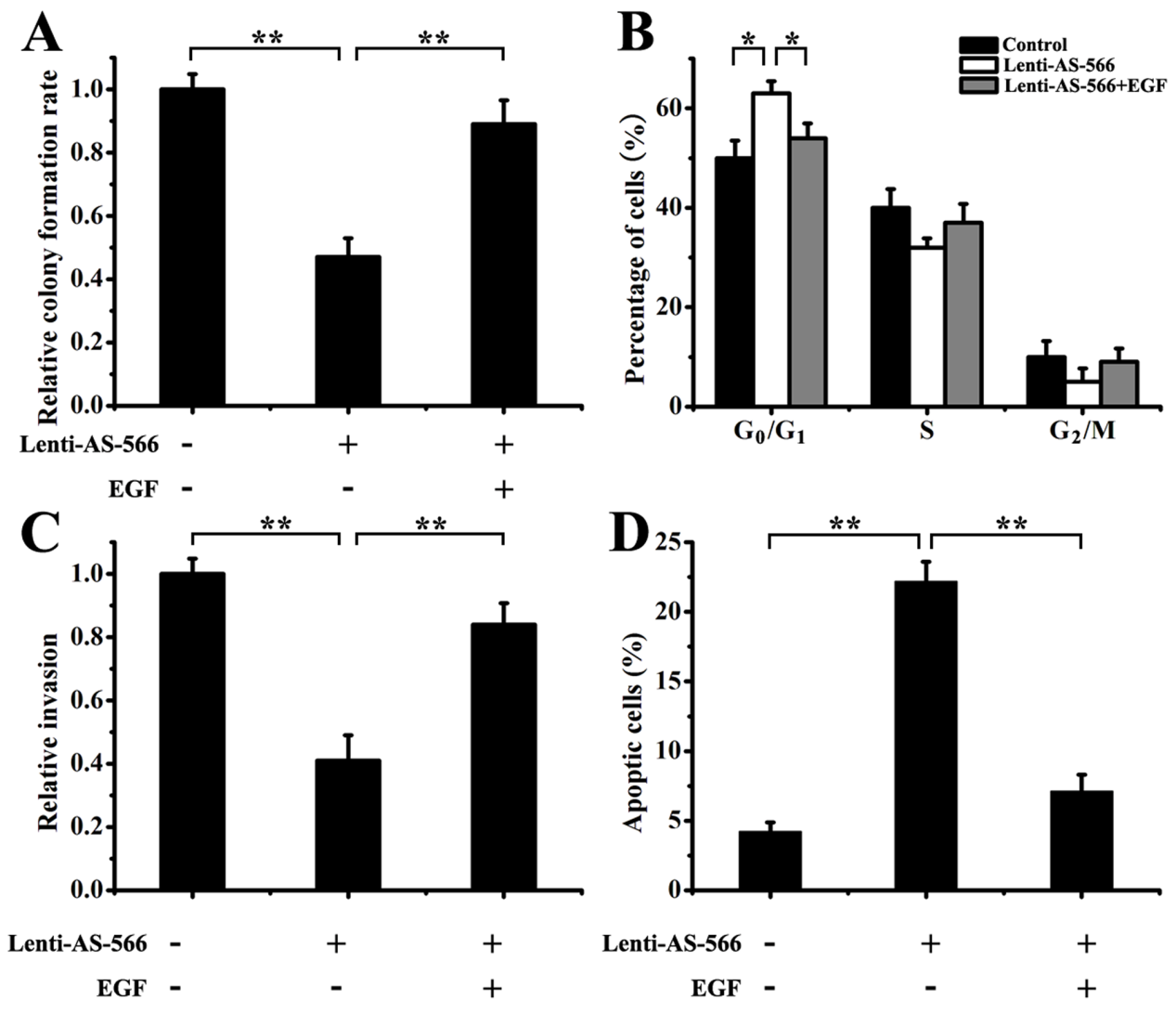


**Additional file 1: Figure S2 - EGF reversed the effects of miR-566 inhibition. (A)** U87 cells were infected or not with lenti-AS-566, 24 h later, EGF (10 ng/ml) was added into the medium. Proliferation **(A)**, cell cycle distribution **(B)**, *in vitro* invasion **(C)**, and caspase3/7 activity **(D)** were evaluated 10 h after EGF treatment. Data in all panels represent the mean ± SD. *, P < 0.05; **, P < 0.01.
